# Adapting Footfall Rhythmicity to Auditory Perturbations Affects Resilience of Locomotor Behavior: A Proof-of-Concept Study

**DOI:** 10.3389/fnins.2021.678965

**Published:** 2021-07-29

**Authors:** Deepak K. Ravi, Caroline C. Heimhofer, William R. Taylor, Navrag B. Singh

**Affiliations:** Institute for Biomechanics, Department of Health Sciences and Technology, ETH Zürich, Switzerland

**Keywords:** time perception, motor control, fall risk, sensorimotor synchronization, sensory cues, recovery potential, movement timing, rhythm perturbations

## Abstract

For humans, the ability to effectively adapt footfall rhythm to perturbations is critical for stable locomotion. However, only limited information exists regarding how dynamic stability changes when individuals modify their footfall rhythm. In this study, we recorded 3D kinematic activity from 20 participants (13 males, 18–30 years old) during walking on a treadmill while synchronizing with an auditory metronome sequence individualized to their baseline walking characteristics. The sequence then included unexpected temporal perturbations in the beat intervals with the subjects required to adapt their footfall rhythm accordingly. Building on a novel approach to quantify resilience of locomotor behavior, this study found that, in response to auditory perturbation, the mean center of mass (COM) recovery time across all participants who showed deviation from steady state (*N* = 15) was 7.4 (8.9) s. Importantly, recovery of footfall synchronization with the metronome beats after perturbation was achieved prior (+3.4 [95.0% CI +0.1, +9.5] s) to the recovery of COM kinematics. These results highlight the scale of temporal adaptation to perturbations and provide implications for understanding regulation of rhythm and balance. Thus, our study extends the sensorimotor synchronization paradigm to include analysis of COM recovery time toward improving our understanding of an individual’s resilience to perturbations and potentially also their fall risk.

## Introduction

The rhythmic alternation of the trunk and limbs is a distinctive, visibly apparent characteristic of human walking. To achieve this, numerous muscles in the body are cyclically activated in a coordinated sequence by neural commands. The precise location of the generation of these neural commands still remains a matter of debate (Dougherty and Ha, 2019), but accumulating evidence suggests the involvement of a distributed network of inter-neurons and motor-neurons in the spinal cord (Takakusaki, 2017; Grillner and El Manira, 2020). Together with descending supraspinal signals and other interacting sensory, vestibular pathways, this movement circuitry contributes to the continuous regulation of our walking rhythm, even in the presence of perturbations (Rossignol et al., 2006; Goulding, 2009; Aoi et al., 2010). Importantly, impaired regulation of rhythmic walking patterns results in either random or stereotypical behavior that limits one’s ability to adapt to perturbations, as observed in subjects with an increased risk of falling (Callisaya et al., 2011; Hamacher et al., 2011), as well as in individuals suffering from movement disorders, e.g., Parkinson’s disease (O’Boyle et al., 1996; Plotnik and Hausdorff, 2008; Ravi et al., 2020) or stroke (Balasubramanian et al., 2009; Krasovsky et al., 2013).

To investigate and identify subtle impairments in rhythmicity and its regulation related to clinical symptomatology, it is necessary to move beyond experiments involving observations during steady state walking, in order to challenge the underlying neuromuscular mechanisms (Full et al., 2002). As a result, one proposed approach has been to exploit the sensorimotor synchronization paradigm (Repp, 2005; Torre et al., 2010), which evaluates a subject’s ability to match the rhythmic oscillations of a limb with an external (often auditory) stimulus, including infrequent temporal perturbations (where beats are presented earlier or later than expected). This paradigm therefore challenges the individual’s inherent rhythmicity during walking and assesses the elicited adaptive motor responses (Chen et al., 2006; Roerdink et al., 2009; Pelton et al., 2010; Wagner et al., 2016; Forner-Cordero et al., 2019). The methodology has, in essence, several positive aspects: the effect of altering rhythms on walking behavior can provide controlled and reproducible access to non-steady-state behavior as encountered in the real-world (e.g., walking on uneven terrains, negotiating obstacles, etc.). Furthermore, listening to music or beats is able to activate motor networks and compensate for impaired internal timing, hence providing a viable vehicle for rehabilitation of movement disorders (Damm et al., 2020). In fact, synchronizing walking to steady metronome beats (without perturbation) has been shown to increase overall balance ability, and be effective for functional locomotor recovery of individuals with stroke (Lee et al., 2018), Parkinson’s disease (Capato et al., 2020), and multiple sclerosis (Maggio et al., 2021).

Early studies investigating walking rhythm deficits using auditory perturbations focused on the modality of temporal correction, i.e., how quickly and/or accurately participants are able to adapt the timing of their footfalls to recover synchronization with the beat after perturbation (Roerdink et al., 2009; Pelton et al., 2010). Here, the main observed parameter is generally the rate or number of walking cycles to achieve convergence to pre-perturbation footfall synchrony. In synchronizing to rhythm-perturbed metronome beats, however, the maintenance of stable whole-body (center of mass, COM) movement patterns can directly influence the timing of footfall correction response to the perturbation. Importantly, the inverse effect of footfall corrections on the dynamic stability of walking remains unaddressed, hence overlooking the critical aspect inherent in this paradigm for understanding an individual’s resilience to rhythm perturbations and falling (Ravi et al., 2021). In this respect, recent empirical work suggests that individuals may prioritize whole-body stability in the stepping process over producing large synchronization corrections at the expense of their balance control (Brauer et al., 2002; Chen et al., 2006; Wright et al., 2014; Roy et al., 2017). However, it is not immediately clear if corrections to footfall timing adjustments are similarly to be expected for the dynamics of the COM, since the position and velocity of the COM is constantly regulated relative to the foot placement to maintain walking balance (Hof et al., 2005, 2010; Wang and Srinivasan, 2014; Ignasiak et al., 2019).

Given the possibility that temporal corrections could destabilize an individual and even induce a fall, it is clearly necessary to better understand how rhythm perturbations affect walking stability. To date, no study has attempted to explicitly test this proposition. Importantly, the extent to which footfall rhythm influences stability may also depend on the rhythm perception ability of the participants [instructing poor rhythm perceivers to synchronize could incur instability (Ready et al., 2019)] and the magnitude of perturbations (Dotov et al., 2019). As more studies including rhythm perturbations are now emerging (Krasovsky et al., 2013; Wright et al., 2017; Geerse et al., 2020; Khan et al., 2020; Nijs et al., 2020), it is timely to identify the governing principles and detail the involvement of dynamic stability during movement adaptation to rhythm perturbations: hence addressing the fundamental question of how footfall rhythmicity interacts with whole-body balance during walking.

A hallmark of successful movement adaptation is faster return to steady state following a perturbation (Hadley et al., 2017). The ability to reliably measure recovery of movement behavior (i.e., resilience) would thus clearly provide an improved understanding of the relationships between task level synchronization outcomes (i.e., number of walking steps to return to footfall synchrony) and dynamic stability of walking (i.e., number of walking steps to return to steady state COM kinematics). In order to address this underlying question, we build upon the unique approach of Ravi et al. (2021) for quantifying an individual’s COM recovery to steady-state patterns after a perturbation. Toward understanding resilience to rhythm perturbations during walking, this pilot study aimed to investigate the relationships between footfall rhythmicity, auditory perturbations, and dynamic stability. To achieve this, the quantification of resilience was applied after young adults were subjected to a beat delayed perturbation in a metronome sequence.

## Materials and Methods

### Study Participants

Twenty healthy young adults [13 males and 7 females; with mean age: 24.9 (standard deviation SD: 2.3) years; height: 1.76 (0.07) m; mass: 72.7 (6.3) kg] with no history of neurological, orthopedic or other disorders that would affect typical walking patterns participated in this study. The protocol was approved by the local institutional review board (protocol #EK 2019-N-178) and all participants provided written informed consent prior to participating, in accordance with the Declaration of Helsinki.

### Experimental Protocol

A single-belt treadmill (h/p/cosmos sports & medical gmbh, Nussdorf, Germany) and a 10-camera 3D optical motion capture system (100 Hz; Vicon Motion Systems, Oxford, United Kingdom) were used to record the participants’ movement patterns. A lower body marker set consisting of 37 reflective markers (see Supplementary Table 1 for anatomical landmarks) was used. Participants wore comfortable shoes and clothing, as well as headphones (Sennheiser HD280 pro, Sennheiser electronic GmbH & Co. KG, Wedemark, Germany) to provide auditory cues and reduce background noise. They were additionally secured with a ceiling-mounted harness (zero bodyweight support) with chest and pelvis straps as a safety precaution against trips and falls.

#### Baseline Condition

**FIGURE 1:**
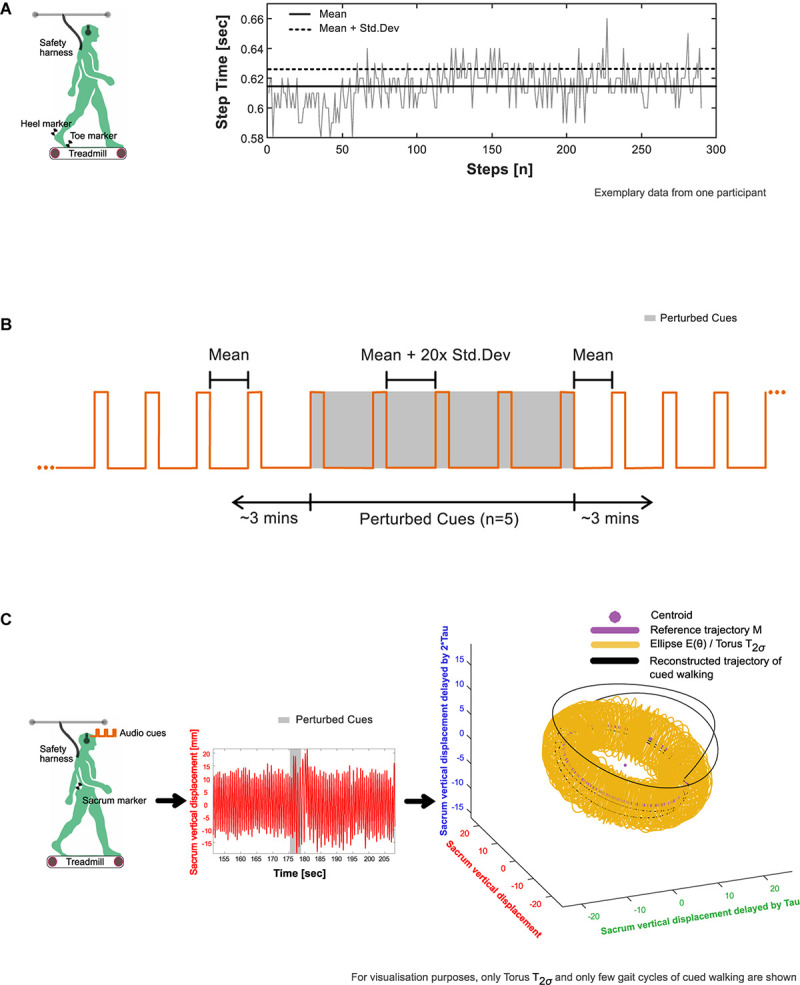
Experimental procedure. **(A)** Extraction of average (mean and standard deviation) step time characteristics from baseline walking 1 (BW1) shown in an exemplary participant. The timing of heel strike and toe off, the events that mark the step time of walking were identified using a custom foot velocity algorithm. **(B)** Generation of subject-specific metronome audio-tracks using BW1 step time characteristics. The inter-beat intervals of the metronome were matched to the mean step time. To induce perturbations, the created track was adjusted by embedding five perturbation intervals, where the inter-beat intervals were increased by 20× the standard deviation of the step time and introduced around 3 min into the track. **(C)** Observation of perturbation response in an exemplary participant’s center of mass movement (approximated in our study using the vertical displacement time series of the sacrum marker) with respect to their steady state boundaries given by the toruses (see section “Determination of Steady State COM Using Baseline Walking 1” in the manuscript for methodological details).

Participants first walked without any auditory stimulus (Baseline walking 1, BW1, 6 min) at their pre-assessed self-selected walking speed. Here, each individual’s baseline step time (defined as the duration from the heel contact of one foot to the heel contact of the contralateral foot, Figure 1A) was evaluated using a custom algorithm based on foot velocity (O’Connor et al., 2007) to inform the auditory conditions for the cued trial. In general, 5-min rest breaks were provided between the trials.

#### Stimulus Preparation

From BW1, a subject-specific metronome audio-track (Supplementary Audio 1) was created in which the inter-beat intervals (IBIs) of the auditory metronome (0.1 s of the musical note A, sine wave with frequency 440 Hz) were matched to the mean step time. To induce rhythm perturbations, the created track was adjusted by embedding five perturbation intervals, with the IBI increased by 20× the standard deviation of the baseline step time (“perturbation magnitude”) and introduced around 3 min into the track (Figure 1B).

#### Cued Condition

Participants were then explicitly instructed to maintain stepping synchronicity with the beats and continue walking normally despite possible alterations to the IBI timing throughout the trial. After providing sufficient time for subjects to practice walking to the provided beat, participants then completed 6 min of cued-walking (CW) listening to the metronome track, which included the planned auditory perturbation.

A further baseline walking trial (BW2, 6 min) without auditory stimulus was then completed by the participants.

### Data Analysis

#### Recovery of COM to Steady-State Patterns

The vertical displacement time series of the sacrum marker [used as a simple approximation of the body COM (Yang and Pai, 2014) and herein referred to as COM] from the three walking trials [formulated as: *X*_*BW*1_(*t*), *X*_*CW*_(*t*), and *X*_*BW*2_(*t*)] were used for further analysis in this study. In order to minimize the start-up effects on walking, the first 5 s of the data were removed from analysis. The data were low pass filtered using a 4th order Butterworth filter with a cut off frequency of 5 Hz and demeaned.

The resultant time series were reconstructed in state space (Figure 1C) using the time delay embedding procedure (Wurdeman, 2016; Supplementary Methods 1). State-space reconstruction of movement time series offers a representation of the underlying dynamics, as well as a geometric illustration of the intrinsic steady-state behavior. An embedding dimension (d) and time lag (τ) were determined from each time series and averaged across the three trials to create the state space vectors [e.g., [*X*_*BW*1_(*t*),  *X*_*BW*1_(*t* + τ), …,*X*_*BW*1_(*t* + (d−1)∗τ)]]. Here, each vector is a state that represents the walking behavior at a specific time, *t*.

In order to determine each subject’s resilience to rhythm perturbations and falling, the recovery of COM kinematics to steady state movement patterns was evaluated [using the methodology developed in Ravi et al. (2021)] as follows:

#### Determination of Steady State COM Using Baseline Walking 1

1. A centroid and reference trajectory (*M*) was first determined on a reduced state space [three dimensions: [*X*_*BW*1_(*t*),  *X*_*BW*1_(*t* + τ),  *X*_*BW*1_(*t* + 2τ)]. The centroid was found by taking the mean of the state space vectors, while *M* was evaluated by fitting an eight-term Fourier model to the reconstructed data. For every state space vector, the corresponding angle relative to the centroid was then calculated using the four-quadrant inverse tangent (Matlab function: “atan2d”).
2. Around *M*, an ellipse at each integer angle (θ) between 0° and 359° was constructed (Figure 1C). Each ellipse was defined using the 50 nearest state space vectors as follows: The length of the semi-major axis of the ellipse was set to the largest standard deviation of the enclosed state space vectors from the three dimensions. The second largest standard deviation gave the length of the semi-minor axis.
3. When schematized, the ellipses adopt the shape of a three-dimensional torus that we term *T*_*1σ*_. Step 2 was repeated to construct *T*_*2σ*_ and *T*_*3σ*_ using two and three times the previously determined standard deviations, respectively.
4. In the context of our analysis, the torus is a steady state region around the reference trajectory to which the COM may return and settle after a perturbation.

#### Evaluation of COM Recovery Using Cued Walking

**FIGURE 2:**
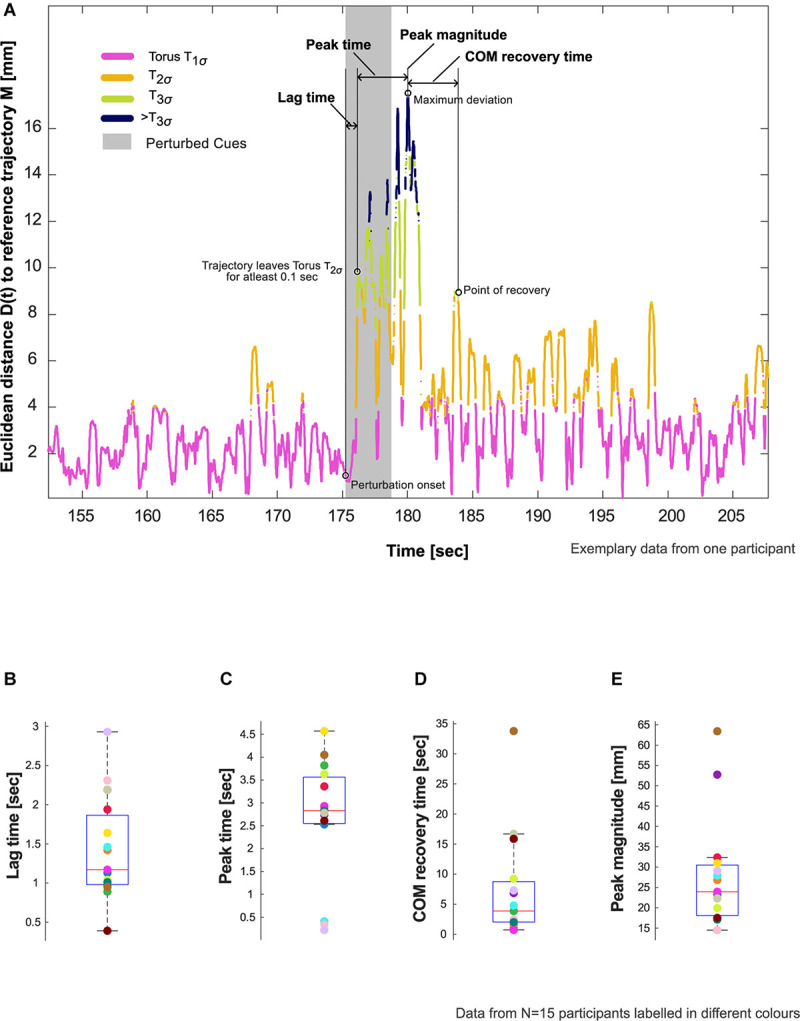
Resilience characteristics. **(A)** Evaluation of lag time, peak time, center of mass (COM) recovery time, and peak magnitude in an exemplary participant who showed deviation from steady state patterns in response to the auditory perturbation. Refer to Section “Evaluation of COM Recovery Using Cued Walking” in the manuscript for the definition and calculation of these characteristics. **(B–E)** Showcasing aggregate data for these characteristics using box plots with median, 25th and 75th percentiles, extreme values, and data from individual participants (*N* = 15).

5. The reconstructed trajectory of *X*_*CW*_(*t*): [*X*_*CW*_(*t*),*X*_*CW*_(t + τ),*X*_*CW*_(t + 2τ)] were now projected onto the tori (Figure 1C). The position of each state space vector was labeled according to the smallest constructed torus, *T*_*1σ*_, *T*_*2σ*_, or *T*_*3σ*_ that enclosed that vector. Subsequently, the Euclidean distance, *D*(*t*), of each state space vector to *M* was calculated (Figure 2A).
6. *D*(*t*) was then parameterized using four variables adapted from Hadley et al. (2017), Figures 2B–E:
**Lag time (s)** – interval between the start of the perturbation to the instant the reconstructed trajectory of *X*_*CW*_(*t*) leaves the torus *T*_*2σ*_ for at least 0.1 s.
**Peak time (s)** – interval after the lag time until the timepoint of maximum deviation of *D*(*t*).
**Peak magnitude (mm)** – magnitude of the maximum deviation.
**COM recovery time (s)** – time interval from the time point of maximum deviation until the point of recovery. The point of recovery of COM was defined as the time point after which the trajectory no longer left the torus *T*_2σ_for five consecutive walking cycles (one walking cycle is equal to the duration between two consecutive heel contacts of the foot), permitting four outliers lasting no more than 0.01 s each (Ravi et al., 2021).

#### Participants’ Aggregate Response to the Auditory Perturbation

In order to understand participants’ aggregate response, the proportion of vector counts that were within (*T*_*1σ*_, *T*_*2σ*_, *T*_*3σ*_) vs. those outside the boundaries (>*T*_*3σ*_) were evaluated using the following additional steps:

7. Step 5 was repeated to evaluate *D*(*t*) for the reconstructed data of BW2:[*X*_*BW*2_(*t*),*X*_*BW*2_(*t* + τ),*X*_*BW*2_(*t* + 2τ)].
8. CW was divided into three phases: (1) start of the walking trial until the onset of the auditory perturbation (**CW_1_**); (2) onset of auditory perturbation until COM recovery (**CW_2_**); (3) point of COM recovery until the end of the trial (**CW_3_**). The vector counts from CW, CW_1_, CW_2_, CW_3_, and BW2 were aggregated for determining the duration within each torus (*T*_*1σ*_, *T*_*2σ*_, or *T*_*3σ*_) and compared.

#### Recovery of Footfall Synchrony to Pre-perturbation Limits

Asynchronies were evaluated as the difference in time between the IBIs (i.e., mean step time from the BW1) and the step times obtained in CW. Pre-perturbation limits were quantified using the SD of the asynchrony from the 10 steps immediately preceding the perturbation [similar to the approach presented by Bank et al. (2011)]. To assess recovery of footfall synchrony following the auditory perturbation, a moving average window analysis was performed. For each window of three steps, mean asynchrony was calculated. The point of recovery of footfall synchronization corresponded to the middle step of the window for which the asynchrony fell within the reference range ± 2 SD of the pre-perturbation asynchrony and stayed within this range for at least eight consecutive windows (corresponding to 5 gait cycles or 10 steps). Synchrony recovery time was calculated as the period between the maximum step time adjustment (given by the peak of the asynchrony after the start of the perturbation) and the point of recovery of footfall synchronization.

### Statistical Analysis

One-way repeated measures ANOVA was used to test for statistical differences in the d and τ values (dependent variables) of each participant between the trials (independent variable). Results were considered to be significant at an alpha of <0.05.

Aggregate data of Lag time, Peak time, Peak magnitude, and COM recovery time were reported as Mean (SD) and visualized using box plots. A stacked bar graph was used to represent relative vector counts between the tori and compared among CW, CW_1_, CW_2_, CW_3_, and BW2.

Mean difference [confidence intervals] between COM recovery time and Synchrony recovery time were estimated. The bootstrap confidence intervals obtained using estimation stats gives a measure of precision and confidence about our estimate (Ho et al., 2019). All analyses were conducted in Matlab (v2020a, The MathWorks, Inc., Natick, MA, United States).

## Results

The average preferred treadmill walking speed was 3.7 (SD: 0.5) Km/h. The participants’ mean step time during BW1 was 0.6 (0.01) s. Accordingly, the average perturbation magnitude and perturbation time were 0.26 (0.06) and 4.3 (0.5) s, respectively.

### Effects of Filtering and Walking Conditions on Tau and Dim

There was no difference in τ and d between unfiltered and filtered data in any of the three walking trials, hence supporting the use of filtered data for state space reconstruction. The one-way ANOVA test confirmed that the differences in τ between walking trials did not reach statistical significance (τ: *F*-ratio value: 0.87, *p*-value: 0.43), while d remained unchanged. The average τ and d across walking trials was found to be 0.2 (0.02) s and 4, respectively.

### Resilience Characteristics

Of the 20 participants analyzed, 5 did not show evidence of the effects of perturbation to COM kinematics (i.e., no deviation from *T*_*2σ*_), and thus were excluded from further analysis. The remaining 15 participants showed an average lag time of 1.4 (0.7) s, peak time of 2.7 (1.3) s, and peak magnitude of 28.0 (13.5) mm. The perturbation resulted in an average COM recovery time of 7.4 (8.9) s (Figure 2B and Table 1).

**TABLE 1:** Demographics, walking, resilience, and asynchrony characteristics.

Demographics (*N* = 20)	
Age (years)	Mean: 24.9 (SD: 2.3)
Height (m)	1.76 (0.07)
Mass (kg)	72.7 (6.3)
Male/female (*n*)	13/7
Treadmill speed (km/h)	3.7 (0.5)
First time on treadmill yes/no (*n*)	4/16
**Baseline walking 1 characteristics (*N* = 20)**	
Step time (s)	0.6 (0.01)
Perturbation magnitude (s)	0.26 (0.06)
Perturbation time (s)	4.3 (0.5)
**Cued walking: resilience characteristics (*N* = 15)**	
Lag time (s)	1.4 (0.7)
Peak time (s)	2.7 (1.3)
Peak magnitude (mm)	28 (13.5)
COM recovery time (s)	7.4 (8.9)
**Proportion of total vector counts within T**_**1**σ_;**T**_**2**σ_; **T**_**3**σ_; > **T**_**3**σ_ **(*N* = 15)**	
CW (%)	74.9 (14); 22.6 (11.6); 1.9 (2.3); 0.6 (0.5)
CW_1_ (%)	77.8 (13.7); 20.8 (11.8); 1.3 (2); 0.1 (0.2)
CW_2_ (%)	25.5 (9.8); 36.6 (13.4); 21.1 (8.6); 16.9 (12.8)
CW_3_ (%)	74.6 (15); 23.8 (12.2); 1.6 (2.8); 0.1 (0.2)
BW2 (%)	75.9 (7); 22.9 (6.4); 1.2 (0.8); 0.1 (0.1)
**Asynchrony characteristics (*N* = 15)**	
Synchrony recovery time (s)	4 (2.1)
Differences in COM recovery time relative to synchrony recovery time (s)	Mean difference: +3.4 [CI: +0.1, +9.5]

*Perturbation magnitude: 20× SD of Step time. Perturbation time: total time for the five perturbed cues, 5× Mean + 20× SD of Step time. SD, standard deviation; COM, center of mass; CW, cued walking; BW, baseline walking; CI, confidence intervals.*

### Synchronization Characteristics

**FIGURE 3:**
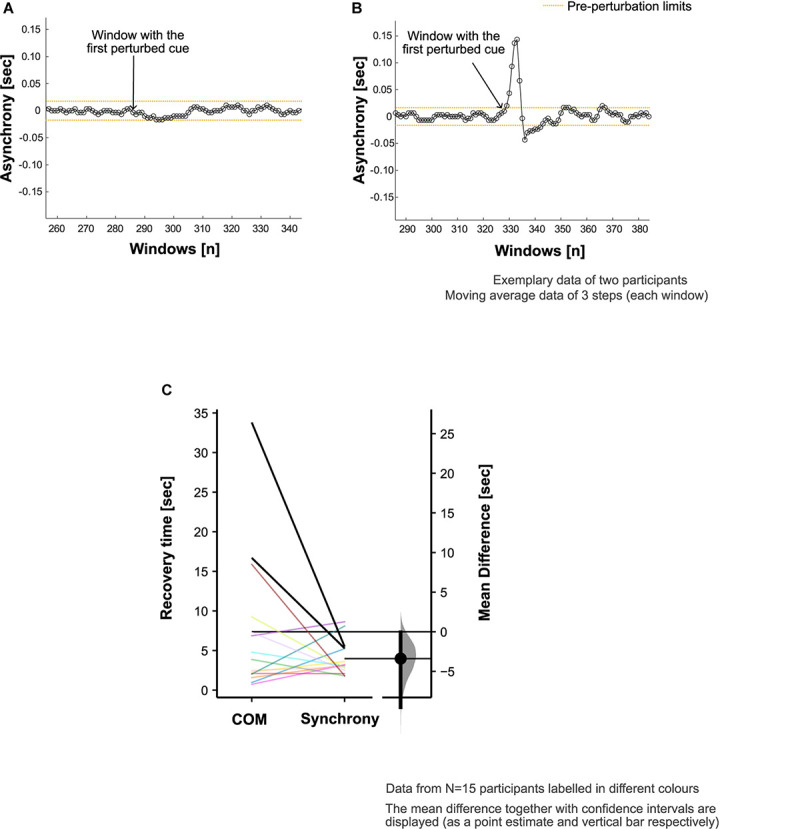
Footfall asynchrony characteristics. Adjustment of step time to align the footsteps with perturbed cues (i.e., synchronization) was achieved in 18 out of 20 participants. Exemplary data are presented to demonstrate the asynchrony (refer to section “Recovery of Footfall Synchrony to Pre-perturbation Limits” in the manuscript for calculation details) characteristics in panel **(A)** a participant who did not adjust their step time to achieve the alignment of the footsteps with the perturbed cues and **(B)** a participant who did synchronize to the perturbed auditory cues. **(C)** Comparison of center of mass (COM) and synchrony recovery times in all the participants who showed deviation from steady state patterns in response to the auditory perturbation (*N* = 15).

The attention and effort to synchronize to the perturbed auditory cues (and step adjustment responses) appeared to induce perturbations to the COM kinematics in the 15 participants who showed COM deviation from steady state patterns (Figures 3A,B). The maximum step time adjustment during CW (given by the peak of the asynchrony, section “Recovery of Footfall Synchrony to Pre-perturbation Limits”) averaged at 0.16 (0.09) s.

In two of the five participants who did not show deviation from steady state, there was no noticeable adjustment of the step time to achieve the alignment of the footsteps with the perturbed cues. The remaining three exhibited no measurable COM deviation from steady state patterns despite synchronizing to the perturbed cues.

In the evaluation of footfall timing adaption to recover synchronization with the beat, the average synchrony recovery time was 4.0 (2.1) s. Overall, the differences in recovery time for COM kinematics relative to footfall synchrony was +3.4 [95.0% CI +0.1, +9.5] s (Figure 3C). However, 7 out of the 15 participants recovered the COM kinematics ahead of the recovery of footfall synchrony.

### Comparison of Vector Counts Between Trials

**FIGURE 4:**
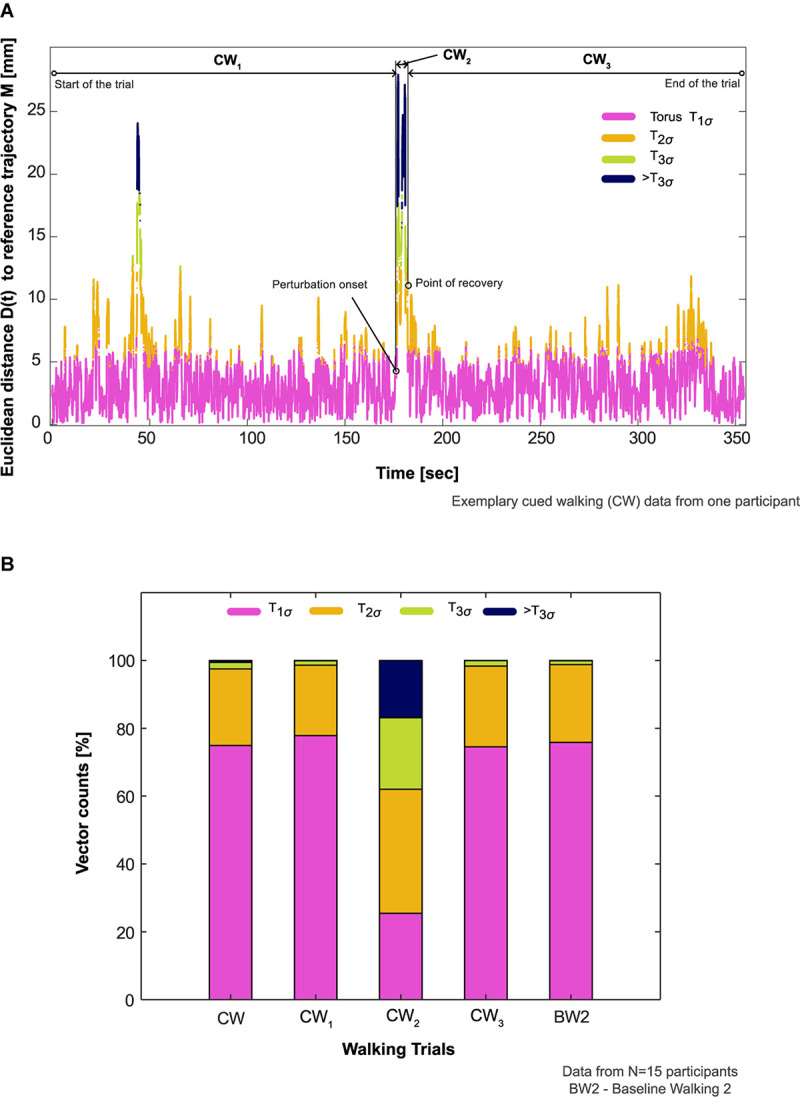
Participants’ aggregate response to the auditory perturbation. **(A)** Comparison of an exemplary participant’s aggregate response in the cued walking (CW) trial during the period of recovery (CW_2_, onset of auditory perturbation until center of mass recovery) to before (CW_1_, start of the walking trial until the onset of the perturbation) and after this period (CW_3_, point of center of mass recovery until the end of the trial). The proportion of vector counts within the steady state boundaries (Torus T_1__σ_, T_2σ_, T_3σ_) and outside (>T_3σ_) determined the aggregate response. **(B)** Comparison of participants’ aggregate response between the different phases of CW, CW itself, and baseline walking 2 (BW2) in all the participants who showed deviation from steady state patterns in response to the auditory perturbation (*N* = 15).

The reconstructed COM trajectory was outside *T*_*3σ*_ on average for 16.8% of CW_2_ (period from onset of auditory perturbation until COM recovery) in comparison to <1% for the rest of the trial (CW_1_ and CW_3_, Figures 4A,B). The differences between CW and BW2 were *T*_*1σ*_ : −0.9%; *T*_*2σ*_ : −0.9%; *T*_*3σ*_ : +0.8%; >*T*_*3σ*_ : +0.5%, Table 1.

## Discussion

The pilot study was designed to investigate whether and how rhythmic auditory perturbations in a sensorimotor synchronization paradigm influence the dynamic stability of walking. This aim was achieved by subjecting 20 healthy young adults to a beat delayed perturbation in a metronome sequence to manipulate their footfall rhythmicity and characterize the resulting COM kinematics. Our results show that in all but five participants, the imposed perturbation modified each individual’s walking rhythm and resulted in COM deviations (average maximum deviation of ∼28 mm) away from steady state patterns. In response to the perturbation, the mean COM recovery time across all participants who showed deviation from steady state (*N* = 15) was 7.4 s (equivalent to approx. 12 steps). Importantly, recovery of footfall synchronization with the metronome beats after perturbation was achieved more rapidly (by 3.4 s) on average compared to COM kinematics. In conclusion, the quantification of an individual’s COM recovery time to steady-state movement patterns after rhythmic auditory perturbations has showcased an experimental framework for assessing an individual’s resilience to rhythm perturbations, and potentially also their fall risk.

Previous studies have shown that synchronizing movement with metronome beats may not be automatic and require some attention and volition (Repp and Keller, 2004; Miyake, 2009; Peper et al., 2012; Terrier and Deriaz, 2013; Mendonca et al., 2014; Hove and Keller, 2015; Leow et al., 2018; Moumdjian et al., 2019). In this respect, metronome beats seem to draw participants’ attention toward the target event in the movement (e.g., footfall) timing, thereby making walking less automatic (Repp, 2005; Peper et al., 2012). This suggests that guided stepping with external auditory cues may require additional frontoparietal structures to be engaged, including networks responsible for attention, e.g., the prefrontal cortex (Wagner et al., 2016, 2019). Interestingly, it has previously been demonstrated that higher attentional demands to perceive a beat and accurately synchronize movement does not negatively influence overall walking stability and balance performance (Nanhoe-Mahabier et al., 2012; Terrier and Deriaz, 2013). Yet this finding does not rule out the risk of instability due to the adjustment of footfall timing at instances when deviations from synchrony occur (naturally: e.g., participants lose attention, desynchronize and attempt to resynchronize, or experimentally: perturbations similar to those imposed in the current study). In line with these expectations, our study substantiates the hypothesis that rhythmic auditory perturbations are able to alter an individual’s stable movement patterns. Here, we observed large inter-individual variability in the maximum deviation (SD: 13.5 mm) and recovery time (SD: 8.9 s) of COM kinematics to relatively small differences in the perturbed time (SD: 0.5 s).

Two participants did not adjust their step time to achieve the alignment of the footsteps with the perturbed cues. It seems that these participants consciously followed and maintained the rhythmic cues before perturbation and not adapted to the variations. Three participants maintained a consistent COM movement pattern within steady state boundaries, despite modifying their footfall rhythm to perturbations. While we cannot be exactly sure how these individuals were able to maintain stable COM kinematics, we would argue that it involves prioritization of different balance strategies or flexible vs. rigid movement responses. On the neurophysiological side, these participants may have allocated less attentional resources toward synchronization (and used the internal cueing from basal ganglia) and more toward the maintenance of balance. Ensuring motor actions in time with perturbed cues may require attention and error-correction processes. The neural mechanisms underlying these processes are continuing to be debated. Neuroimaging studies have linked them to a broader network of brain areas including the auditory cortex, basal ganglia, cerebellum, pre-motor, and Supplementary Motor Areas (Grahn and Brett, 2007; for a review, see also Koshimori and Thaut, 2018; Damm et al., 2020). Further, a recent electrophysiological study suggest signatures of step adaptation to auditory perturbations in the cortical beta activity: beta band supression in the central and parietal cortex and an increase of beta power in the prefrontal regions (Wagner et al., 2016). The authors suggest that the former may be involved in the readiness and voluntary execution of movements and the latter may allow the cognitive flexibility to adapt the movements. And in so doing, these research promises to enrich our understanding of the neural mechanisms underlying movement adaptation to auditory perturbations.

Prior research advocates a close connection between the process of synchronization correction and maintaining stable movement patterns during adaptation to rhythm perturbations (Chen et al., 2006; Wright et al., 2014; Hove and Keller, 2015). When a perturbation occurs in the auditory sequence, a subject’s behavioral response to restore synchrony with the cues may be constrained by the position of their COM relative to their feet to maintain balance and prevent falling. In the present study, we additionally tested whether the participants prioritized the recovery of COM kinematics over footfall synchronization after perturbation. However, we found no substantial evidence for this expectation, i.e., no significant differences between COM and synchrony recovery times. A caveat of these findings may be the young and healthy composition of our cohort, as it has been shown that such subjects may not be as susceptible to dual-tasking interference as older adults and clinical populations (Brauer et al., 2001, 2002). Here, older populations might prioritize COM kinematics over task synchrony in order to reduce their propensity to fall (Bleom et al., 2006). While these issues remain to be elucidated in future studies, the findings may have important implications regarding task prioritization in real-life walking scenarios.

Neural feedback mechanisms for sensing and responding to rhythm perturbations naturally involve latencies (Wagner et al., 2019; Zhang et al., 2020). When combined with timing constraints to integrate information from sensory and motor systems in higher brain centers for movement planning and execution, such latencies may be critical for the recovery time of movement to perturbations (Repp and Su, 2013; Hove et al., 2014; Daley, 2018; Koshimori and Thaut, 2018). Our previous experimental work (Ravi et al., 2021) and the results from the current study have largely detailed how humans recover COM kinematics (i.e., resilience) gradually to steady-state patterns in subsequent steps after perturbation. One might ask, therefore, whether individuals are unstable during the apparently long period of recovery. To elucidate this, we analyzed each participant’s COM trajectory composition within different steady state boundaries and found that movement patterns during the period of recovery (CW_2_, Figure 4A) exhibited a noticeable proportion of time outside *T*_*3σ*_ (16%, Figure 4B) in comparison to other phases of CW.

The magnitude of perturbations to the auditory stimuli used in the majority of published literature to date has been selected somewhat arbitrarily and discordantly: phase shifts of 50 ms (Chen et al., 2006); 60° (Roerdink et al., 2009; Nijs et al., 2020); 100 ms (Wright et al., 2014); and 15% of step cycle (Khan et al., 2020). In the present study, we perturbed the metronome cues based on each individual’s step time variability (measured in standard deviations). This approach is arguably less susceptible to participant bias due to its insight into each individual’s walking performance, rather than at arbitrarily chosen levels. However, further study should be carried out to investigate the advantages and disadvantages of this approach. Future work must also address the differences in COM recovery time between lengthening (as used in the current study) and shortening the IBI of the perturbed cues. Encouragingly, there is previous evidence that participants make larger footfall corrections (that could incur larger instability) when the perturbation IBIs are shortened as opposed to lengthening the intervals (Wright et al., 2014).

It has been suggested that individuals rely on cycle-to-cycle corrections to maintain coordination with an auditory stimulus (Vaz et al., 2019). As such, studies typically analyze only discrete events in the walking cycle (e.g., heel contact), but corrective adjustments are likely to occur continuously throughout different phases of a gait cycle. Here, the analysis of footfall synchrony recovery time could be further extended to characterize the distribution of timing correction across a walking cycle. When combined with our analyses to quantify resilience to perturbations, such approaches provide a valuable insight into gait phase-specific variation in balance response and its interaction with external sensory cues and perturbations. Moreover, these approaches could lay the foundations for understanding rhythm deficits in neuromotor pathologies and rehabilitation, and thereby support clinical decision making.

A number of limitations of this pilot investigation should be acknowledged. First, our experimental protocol was limited to recording only a few body segments. Notably, we could not determine the body’s actual COM, but rather used only an approximation based on the sacral marker. Additionally, it was not possible to provide a characterization of upper body rhythmic movement response (i.e., timing of arm swing or head bobbing) to auditory stimulation and perturbation. Second, we did not collect subjective feedback about the experiment. In this study, two participants missed the perturbed cues, despite prior instruction to synchronize to the auditory cues. While this was unexpected, post-experiment feedback would have allowed us to better understand whether these participants perceived the perturbation in the auditory cues or misunderstood the instructions. Third, while the minimum number of dimensions required to form properly a state-space of the COM was evaluated to be 4, we used only three dimensions in the current study. This is a limitation of our 3D torus-based approach but the sensitivity of our findings to such differences need further exploration. Fourth, we could not align the audio track and motion capture data in the time axis, because we failed to account a temporal delay in one of the data streams. However, this temporal delay did not affect the analyses and results of the present study.

In summary, locomotion in complex, dynamic real-world environments is an integral part of daily life of humans. While walking in such environments, individuals modulate their footfall timing and rhythmicity to maintain the body’s COM, which is a critical factor in their successful and continuous ambulation (i.e., fall avoidance). The present study extended the sensorimotor synchronization paradigm to include analysis of COM recovery time and has provided a novel framework for improving our understanding of an individual’s resilience to perturbations. It also provides a starting point in the use of these techniques toward understanding an individual’s ability to avoid falls. At a fundamental level, we show not only differences between COM and synchrony recovery times, but that even young adults took up to 12 steps to recover stable movement patterns from a relatively innocuous perturbation.

## Data Availability Statement

The raw data supporting the conclusions of this article will be made available by the authors, without undue reservation. The codes to reproduce the central findings in this study (COM recovery time after auditory perturbations) is publicly available at: https://github.com/laboratory-of-movement-biomechanics-eth/locomotor-resilience.

## Ethics Statement

The studies involving human participants were reviewed and approved by ETH Zürich Ethics Commission. The patients/participants provided their written informed consent to participate in this study.

## Author Contributions

DR and CH conceived and designed the study. NS and WT supervised the project and provided critical opinions as subject experts. CH performed the data collection. DR and CH did the subsequent data analysis. DR, WT, and NS drafted the manuscript. WT is the guarantor. All the authors reviewed and approved the final manuscript for submission.

## Conflict of Interest

DR was supported by SERI Switzerland. The funder was not involved in the study design, collection, analysis, interpretation of data, the writing of this article or the decision to submit it for publication. The remaining authors declare that the research was conducted in the absence of any commercial or financial relationships that could be construed as a potential conflict of interest.

## Publisher’s Note

All claims expressed in this article are solely those of the authors and do not necessarily represent those of their affiliated organizations, or those of the publisher, the editors and the reviewers. Any product that may be evaluated in this article, or claim that may be made by its manufacturer, is not guaranteed or endorsed by the publisher.
