# Supplementary material for: Adapting Footfall Rhythmicity to Auditory Perturbations Affects Resilience of Locomotor Behavior: A Proof-of-Concept Study
Source: Front Neurosci. 2021 Jul 29;15:678965. doi: 10.3389/fnins.2021.678965 (PMC8358836; doi:10.3389/fnins.2021.678965)
Supplement: Supplementary file 1 [file Data_Sheet_1.zip › Supplementary Materials/Supplementary Material.docx]

**Supp. Table 1. Anatomical landmarks of the marker set used**

| **Name** | **Segment** | **Location** |
| --- | --- | --- |
| RTO1 | Foot - right | Caput metatarsale I |
| RTO3 | Foot - right | Metatarsale III |
| RTO5 | Foot - right | Caput metatarsale VI |
| RHEE | Foot - right | Calcaneus |
| RMMA | Shank - right | Medial malleolus |
| RLMA | Shank - right | Lateral malleolus |
| RTMT | Shank - right | unspecified Tibia, ventral |
| RTLF | Shank - right | unspecified Fibula, lateral |
| RTTT | Shank - right | Tuberositas tibea |
| RTIB | Shank - right | Caput fibulae |
| RMCO | Thigh - right | Medial femur epicondylus |
| RLCO | Thigh - right | Lateral femur epicondylus |
| RTFR | Thigh - right | unspecified Femur, front distal |
| RTLL | Thigh - right | unspecified Femur, lateral distal |
| RTLH | Thigh - right | unspecified Femur, lateral proximal |
| RASI | Pelvis | Spina illiaca ant. sup. – right |
| RTMS | Pelvis | Iliaca – right |
| RPSI | Pelvis | Spina illiaca post. Sup – right |
| SACR | Pelvis | Sacrum |
| LPSI | Pelvis | Spina illiaca ant. sup. – left |
| LTMS | Pelvis | Iliaca - left |
| LASI | Pelvis | Spina illiaca post. Sup - left |
| LTO1 | Foot - left | Caput metatarsale I |
| LTO3 | Foot - left | Metatarsale III |
| LTO5 | Foot - left | Caput metatarsale VI |
| LHEE | Foot - left | Calcaneus |
| LMMA | Shank - left | Medial malleolus |
| LLMA | Shank - left | Lateral malleolus |
| LTMT | Shank - left | unspecified Tibia, ventral |
| LTLF | Shank - left | unspecified Fibula, lateral |
| LTTT | Shank - left | Tuberositas tibea |
| LTIB | Shank - left | Caput fibulae |
| LMCO | Thigh - left | Medial femur epicondylus |
| LLCO | Thigh - left | Lateral femur epicondylus |
| LTFR | Thigh - left | unspecified Femur, front distal |
| LTLL | Thigh - left | unspecified Femur, lateral distal |
| LTLH | Thigh - left | unspecified Femur, lateral proximal |

**Supp. Audio 1. Cueing stimuli with perturbation from an example participant (.wav)**

**Supp. Method 1: Time delay embedding:**

State-space reconstruction is a powerful approach for estimating the underlying dynamics and structure of a time series. It involves the analysis and geometrical representation of how a dynamical system change over time in an n-dimensional ‘state space’ where n is the number of state variables. The first step is to specify the state variables such as displacement and velocity that completely define the state of the system. Oftentimes, it is difficult to know and measure all the possible state variables of a system. In such cases, state spaces may be defined using a procedure called time delay embedding, which allows unfolding the dynamics of a system from any single variable and its time delayed copies.

An embedding dimension and time lag are required for using this procedure. ‘*The dimension of the space that contains the true structure of a dynamical system is called the embedding dimension’ (Wurdeman 2016). ‘Time lag gives the “right” amount of information about the system needs to be identified’ (Wurdeman 2016).*

We used the most commonly used method - the false nearest neighbor algorithm to find an appropriate embedding dimension and the average mutual information to find an appropriate time lag. These algorithms are available in the built in Matlab function ‘phaseSpaceReconstruction.m’ to perform state space reconstruction.

**References:**

Wurdeman, S. R. (2016). State-Space Reconstruction. Nonlinear Analysis for Human Movement Variability. N. Stergiou, CRC Press Taylor & Francis Group**:** 55-82.

<https://ch.mathworks.com/help/predmaint/ref/phasespacereconstruction.html>
